# TGF-*β* Inhibitor SB431542 Promotes the Differentiation of Induced Pluripotent Stem Cells and Embryonic Stem Cells into Mesenchymal-Like Cells

**DOI:** 10.1155/2018/7878201

**Published:** 2018-07-02

**Authors:** Alessander Leyendecker Junior

**Affiliations:** Monash Health, Monash University, Clayton, Melbourne, VIC, Australia

## Abstract

Due to their potential for tissue engineering applications and ability to modulate the immune system and reduce inflammation, mesenchymal stem cells (MSCs) have been explored as a promising option for the treatment of chronic diseases and injuries. However, there are problems associated with the use of this type of cell that limit their applications. Several studies have been exploring the possibility to produce mesenchymal stem cells from pluripotent stem cells (PSCs). The aim of these studies is to generate MSCs with advantageous characteristics of both PSCs and MSCs. However, there are still some questions concerning the characteristics of MSCs derived from the differentiation of PSCs that must be answered before they can be used to treat diseases and injuries. The objective of this study was, therefore, to determine if PSCs exposed to SB431542, a TGF-*β* inhibitor, are able to differentiate to MSCs, judging by morphology, expression of mesenchymal and pluripotent stem cell markers, expression of pluripotency-related genes, and ability to differentiate to osteocytes and adipocytes. The results obtained demonstrated that it is possible to induce the differentiation of both embryonic stem cells and induce pluripotent stem cells into cells with characteristics that highly resemble those from MSCs through the inhibition of the TGF-*β* pathway.

## 1. Introduction

Stem cells are undifferentiated cells that have an extraordinary ability to self-renew via cell division and differentiate into one or more specialized types of cells [1]. Because of their great potential in tissue engineering, they have been intensively studied as alternatives for the treatment of a wide variety of diseases and injuries. According to their origin, stem cells can be classified as embryonic stem cells (ESC), adult stem cells, and induced pluripotency stem cells (iPSCs). ESCs are obtained from the internal mass of a blastocyst and, because of their ability to originate all the cells of the embryo proper, are classified as pluripotent stem cells (PSCs) [2]. Adult stem cells, on the other hand, are found in most adult tissues and are classified as multipotent stem cells as they are capable of giving rise to a more restricted variety of cells when compared to PSCs. Finally, iPSCs are pluripotent stem cells obtained through genetic reprogramming of adult cells [3].

Mesenchymal stem cells (MSCs) are multipotent cells that have the ability to differentiate into mesodermal cell lines, including chondroblasts, osteoblasts, and adipocytes [4]. This type of stem cell, despite being classically obtained from the bone marrow [5], can also be isolated from a number of neonatal and adult tissues, including dental pulp [6], orbicularis oris muscle [7], and fat [8]. When cultured, these cells can be easily identified by their elongated and fusiform fibroblast-like morphology, with large, oval, euchromatic, and central nuclei and abundant cytoplasm [9]. In 2006, the International Society for Cellular Therapy (ISCT) [10] established that the presence of three basic characteristics must be evidenced so that a culture of cells isolated from adult tissues can be effectively classified as being a culture of MSCs. First, MSCs must be able to adhere to the plastic present in cell culture containers. In addition, at least 95% of the cell population isolated and expanded in culture must express the mesenchymal antigens CD29, CD44, ecto-5-nucleosity (CD73), Thy-1 (CD90), and endoglin (CD105), and no more than 2% of the cells in this population should express the hematopoietic markers CD14, CD19, CD34, CD45, and HLA-DR. Finally, MSCs should be able to differentiate into osteoblasts, chondroblasts, and adipocytes in vitro under specific culture conditions [10].

Because of its ability to integrate and differentiate into cells of an injured tissue, MSCs have been studied as a promising tool for cellular therapies and bone [11, 12], cartilage [13], and tendon [14] tissue bioengineering. However, many of the therapeutic properties of MSCs have been attributed to the paracrine and endocrine action of secreted factors. Notably, MSCs have been shown to be capable of supporting the maturation and proliferation of hematopoietic cells and to migrate to an area of tissue injury, recruit tissue-specific progenitor cells [15], and regulate the immune response through the secretion of immunomodulatory cytokines and growth factors (such as PGE2, IL-4, IL-6, IL-10, TGF-*β*, HGF, and IDO) and microvesicles containing a variety of bioactive molecules such as enzymes, coding and noncoding RNAs, and heat shock proteins [16]. Positive results from preclinical trials and the demonstration of immunomodulatory properties of MSCs in in vitro experiments led to a rapid increase in the number of clinical trials in which the therapeutic potential of these cells was evaluated for the treatment of a variety of diseases. It is expected that, in the future, it will be possible to isolate MSCs from a number of tissues, expand them in culture, and produce billions of cells that will be administered locally or intravascularly for the treatment of diseases. Examples of diseases in which the therapeutic use of MSCs has been evaluated in clinical and preclinical trials and has shown promising results include acute myocardial infarction [17], systemic lupus erythematosus [18], rheumatoid arthritis [19], Crohn's disease [20], acute lung injury [21], autoimmune-associated lung fibrosis [22], chronic obstructive pulmonary disease [23], liver cirrhosis [24], multiple sclerosis [25], amyotrophic lateral sclerosis [26], and type I diabetes [27].

Despite all the potential that the clinical use of this type of stem cell represents, there are some important problems associated with the current methods of obtaining and culturing these cells that substantially limits their use in cell therapies. First, only an extremely rare subpopulation of MSCs isolated from adult tissues retains, when cultured, their proliferative capacity and their differentiation potential in multiple cell lines. Moreover, cells isolated from different tissues, derived from donors of different ages or maintained under different cell culture conditions, present considerable differences with respect to their proliferative and differentiation potentials [28]. Finally, methods of obtaining MSCs from adult tissues are generally invasive and the cells isolated usually acquire phenotypic, biochemical, molecular, and functional changes when cultured for long periods, resulting in eventual replicative senescence [29]. On the other hand, PSCs have the ability to differentiate into the three germ layers (ectoderm, endoderm, and mesoderm) and can be maintained in culture indefinitely without showing any significant signs of replicative senescence or loss of potentiality. With this in mind, several research groups have focused their efforts on achieving the most direct and efficient differentiation of PSCs into MSCs [30–32] in order to generate a virtually unlimited source of MSCs that are both safe and functional for later use in cell therapies.

Despite all these unanswered questions, there are numerous potential benefits that the production of a virtually unlimited source of MSCs from the differentiation of PSCs represents. MSCs produced by this method can be used not only to treat a wide variety of diseases but also to model diseases resulting from genetic disorders that have an effect on MSCs or on cells derived from their differentiation and to test medicaments which may, in the future, be used to treat these disorders. It is expected that, eventually, iPSCs may be produced from reprogramming adult cells of a given individual, differentiated into MSCs, expanded in vitro, and used in the treatment of the individual (autologous transplant) or in the treatment of others (allogeneic transplantation). Furthermore, cells that present some genetic anomaly may have this anomaly corrected through genetic therapy techniques and be transplanted again to the patient. With these advantages in mind, this study is aimed at generating MSCs from ESCs (ESC-MSC) and iPSCs (iPSC-MSC) and investigating the differences between BM-MSCs and ESC-MSCs and iPSC-MSCs generated.

## 2. Methodology

### 2.1. Human Pluripotent Stem Cell Culture

In order to expand pluripotent cells under human-like conditions, three distinct human embryonic stem cell lines (Genea 02, H9, and H9 OCT4 mCHERRY) and one induced pluripotent stem cell line (NF1) were cultured in Geltrex-coated T25 (25 cm^2^ area culture flask) tissue culture flasks under feeder-free and xeno-free conditions. Cultured pluripotent stem cells were split to new Geltrex-coated flasks when the confluency was around 80% (approximately 3–4 days) in order to avoid cell differentiation and senescence. A complete medium change was performed every day, and the cells were split again once a confluency of 80% was reached. All undifferentiated iPSC and human ESC colonies were maintained in Essential 8 medium (E8 medium; Life Technologies) and incubated at 37°C in 5% CO_2_ humidified incubators. iPSCs and ESCs were maintained in T25 flasks and transferred to T75 flasks for mesenchymal differentiation, RT qPCR, and FACS analysis.

### 2.2. Bone Marrow Mesenchymal Stem Cell Culture

Mesenchymal stem cells stored in vapour phase nitrogen were thawed and maintained in Geltrex-coated T75 flasks in 10% FBS-MPC Growth MEM media (Lonza). A complete medium change was performed every 3–4 days, and the cells were reseeded to new T75 flasks at a density of 2000 cells per cm^2^ once they reached around 90% confluency. BM-MSCs were maintained at 37°C in a 5% CO_2_ humidified incubator until use.

### 2.3. Inducing Differentiation of ESCs and iPSCs into MSC-Like Cells

A method proposed by Chen et al. [33] was used, with some modifications, in order to induce differentiation of ESCs and iPSCs to MSC-like cells. All cells were initially maintained in complete E8 medium on Geltrex-coated T75 flasks as large colonies at high confluence as described above. Once a confluency between 80% and 90% was reached, the complete E8 medium was changed to an inhibitor differentiation medium that consists of Essential 6 medium (E6 medium; Life Technologies) supplemented with the TGF-*β* pathway inhibitor SB431542 (Sigma-Aldrich) at 10 *μ*M and replaced daily with a fresh one for 10 days. The difference between the E8 and E6 media is the absence of bFGF and TGF-*β* in the E6 composition. Pictures were also taken daily using the Leica DV100 digital camera attached to the inverted Leica DMR fluorescent microscope (Leica, Switzerland) in order to evaluate the morphological alterations in the pluripotent stem cell colonies during the differentiation process. Images were collected using Analysis software (Olympus).

All pluripotent stem cells induced to differentiate to MSC-like cells were split to new T75 Geltrex-coated flasks after 10 days of incubation in E6 SB431542 inhibitor differentiation medium (MP0). The ESC-MSCs and iPSC-MSCs were then transferred to T75 flasks as single cells, reseeded at a density of 40,000 cells per cm^2^ in 10% FBS-MPC Growth MEM media (Lonza), and maintained at 37°C in a 5% CO_2_ humidified incubator. Cultured ESC-MSCs and iPSC-MSCs were split using the same method after one week (MP1) and reseeded at 20,000 cells per cm^2^ in 10% FBS-MPC Growth MEM media in the second mesenchymal passage (MP2) and at 10,000 cells per cm^2^ in subsequent passages (MP3, MP4, etc.).

### 2.4. Cell Surface Marker Analysis

The expression of specific extra- and intracellular molecular markers was analysed by flow cytometry in the following cell populations: three undifferentiated ESC lines (GENEA 02, H9, and H9 OCT4 mCHERRY) and one iPSC line (NF1); cells derived from the differentiation of all pluripotent stem cells into MSC-like cells (GENEA 02-MSC, H9-MSC, H9 OCT4 mCHERRY-MSC, and NF1-MSC) after 10 (MP0), 17 (MP1), and 24 (MP2) days of directed mesenchymal differentiation and bone marrow-derived hMSCs at any passage. All cells were incubated with fluorochrome-conjugated antibodies detecting cell surface (CD29, CD44, CD90, CD9, SSEA3, Tra 1-60, and HSP90*β*) and intracellular (Oct-4) markers in order to compare and characterize them according to molecules present on their membrane or intracellularly. A sample of unstained cells were also prepared for both intracellular and cell surface staining experiments in order to detect any background staining or autofluorescence innate to the cells. For negative control, an isotype control was used for every immunoglobulin tested. For data collection, cells were passed through an LSR II flow cytometer equipped with BD FACSDiva software (BD Biosciences). The data analysis was conducted using FlowJo software (Tree Star Inc.). Forward- and side-scatter plots were used in order to eliminate doublets and debris from the analysis. Dead cells were excluded from the analysis by selection against propidium iodide. Positive expression of a marker was assessed by comparison of the distribution of events given by the isotype-negative control compared with the stained sample. Gates representing the positive expression of the marker were determined by exclusion of greater than 99% events appearing in the isotype control pattern.

### 2.5. Quantitative Real-Time Polymerase Chain Reaction (RT-qPCR)

Quantitative polymerase chain reaction (qPCR) was conducted in order to compare and characterize BM-MSCs, ESCs, ESC-MSCs, and iPSC-MSCs according to mRNA expression levels. The expression of the pluripotency-associated Oct-4 gene was analysed by qPCR before and after the mesenchymal differentiation process.

### 2.6. Osteogenic Differentiation Assay

In order to proceed with the osteogenic differentiation assay, 3.1 × 10^3^ BM-MSCs, ESC-MSCs, and iPSC-MSCs were seeded per cm^2^ of tissue culture in 24-well plates. Cells were then allowed to adhere to the culture surface for 4 to 24 hours in MPC Growth MEM media (Lonza) at 37°C, in a humidified atmosphere of 5% CO_2_. The osteogenesis was then inducted by replacing the MPC Growth MEM media with osteogenesis induction medium. The osteogenesis induction medium was completely replaced with fresh osteogenesis induction medium every 3–4 days for 4 weeks. The noninduced control cells were fed with MPC Growth MEM media on the same schedule. Photos were taken using a microscope every 3–4 days in order to evaluate the osteogenic differentiation process. The amount of calcium produced during the osteogenic differentiation of ESC-MSCs and iPSC-MSCs was quantified by spectrophotometry and compared with BM-MSCs in order to evaluate the effectiveness of the process.

### 2.7. Adipogenic Differentiation Assay

In order to proceed with the adipogenic differentiation assay, 2.1 × 10^4^ BM-MSCs, ESC-MSCs, and iPSC-MSCs were seeded per cm^2^ of tissue culture in 24-well plates. Cells were maintained in MPC Growth MEM media (Lonza) at 37°C, in a humidified atmosphere of 5% CO_2_ until the confluence was reached. The cells were fed by completely replacing the MPC Growth MEM media every 3–4 days with fresh MPC Growth MEM media. Once 100% confluence was reached, adipogenesis was inducted by replacing the MPC Growth MEM media with adipogenesis induction medium and replacing it with adipogenesis maintenance medium (Lonza) after 4 days of culture. Three cycles of induction/maintenance are necessary in order to stimulate optimal adipogenic differentiation. Each cycle consists of culturing the cells in adipogenesis induction medium for 4 days at 37°C in a 5% CO_2_ incubator followed by 3 days of culture in adipogenic maintenance medium at 37°C in a 5% CO_2_ incubator. After 3 complete cycles of induction/maintenance, the cells were cultured for 7 more days in adipogenic maintenance medium. The cells were fed by replacing the adipogenic maintenance medium every 2–3 days with fresh adipogenic maintenance medium. The noninduced control cells were fed with MPC Growth MEM media on the same schedule. Photos were taken using a microscope every 3–4 days in order to evaluate the adipogenic differentiation process. The adipogenic differentiation process could be observed by the accumulation of lipid-rich vacuoles within cells. The amount of lipid produced during the differentiation process was assessed by the staining with AdipoRed Kit (Lonza). The fluorescence values were then read on the EnSpire Machine fluorimeter (excitation 485 nm, emission 572 nm). The amount of fluorescence detected is directly proportional to the amount of lipid produced and to the effectiveness of the adipogenic differentiation.

### 2.8. Statistical Analysis

Data analysis was performed using GraphPad Prism 5.0 (GraphPad Software). Data were analysed by standard deviation to assure the reproducibility of results and represented as error bars in each figure.

## 3. Results

### 3.1. Morphological Changes during Differentiation

**Figure 1:**
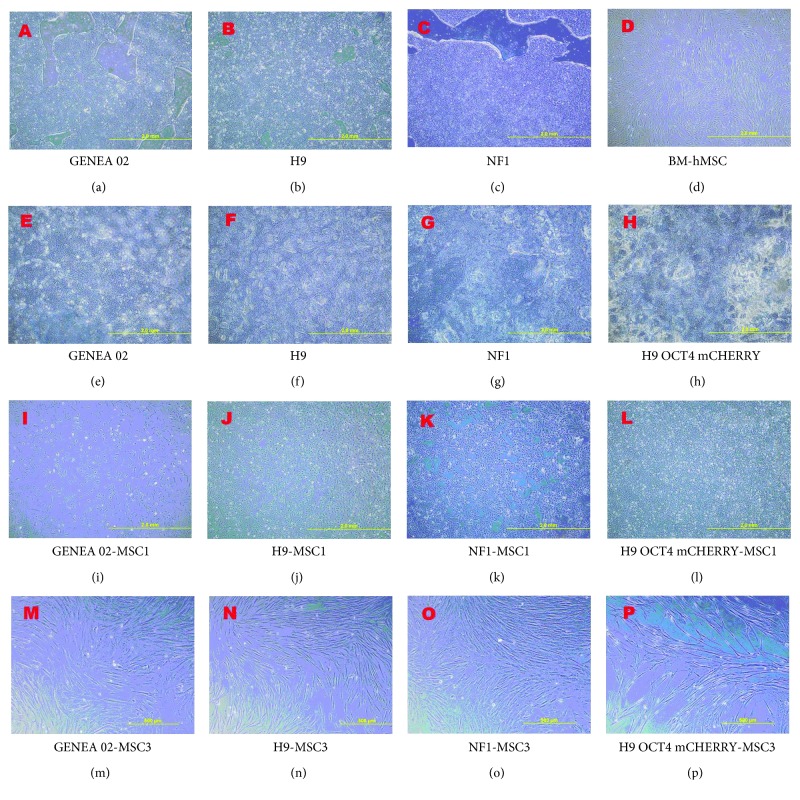
Morphological characteristics of GENEA 02, H9, NF1, and BM-MSCs before and during the differentiation process. (a, b, c) Typical circular and flat colonies with a well-defined border observed in undifferentiated human embryonic stem cells and induced pluripotent stem cells before the differentiation process. (d) Typical spindle-shaped morphology of bone marrow-derived human mesenchymal stem cells. (e, f, g, h) Regions of SB-induced differentiation were apparent as cells develop an enlarged, flattened epithelial-like morphology in a circular conformation after 10 days of SB431542 exposure. (i, j, k, l) An intermediate morphology was observed on GENEA 02-MSC1, H9-MSC1, and NF1-MSC1 cells while H9 OCT4 mCHERRY-MSC1 still appeared as large and flat cells after one passage in MPC Growth MEM media. (m, n, o, p) A spindle-shaped morphology similar to that of BM-hMSCs was observed in all cell lines examined after three passages in MPC Growth MEM media.

In the absence of SB431542, undifferentiated iPSCs and ESCs cultured on Geltrex in complete E8 medium appeared as circular and flat colonies with a well-defined border (Figures 1(a)–1(c)). The exposure to SB431542 for 10 days induced differentiation in both human ES- and iPS-derived cells as indicated by the presence of a monolayer of large cuboidal epithelial-like cell colonies in a circular conformation (Figure 1(e)–1(h)). Seven days after passage into MPC Growth MEM media (day 17 of directed mesengenic differentiation), an intermediate morphology was observed on GENEA 02, H9, and NF1 cells (Figure 1(i)–1(k)) while H9 OCT4 mCHERRY cells were still large and flat (Figure 1(l)). The morphological alteration from circular and flat colonies with a well-defined border observed before differentiation to spindle-shaped cells was observed after 24 days of directed mesengenic differentiation on GENEA 02, H9, and NF1 cells and after 31 days on H9 OCT4 mCHERRY cells (Figure 1(m)–1(p)).  Figure 1 contains some pictures that were taken during the process of differentiation in order to evaluate the morphological alterations in the pluripotent stem cell colonies during the process.

### 3.2. Cell Marker Analysis

**Figure 2:**
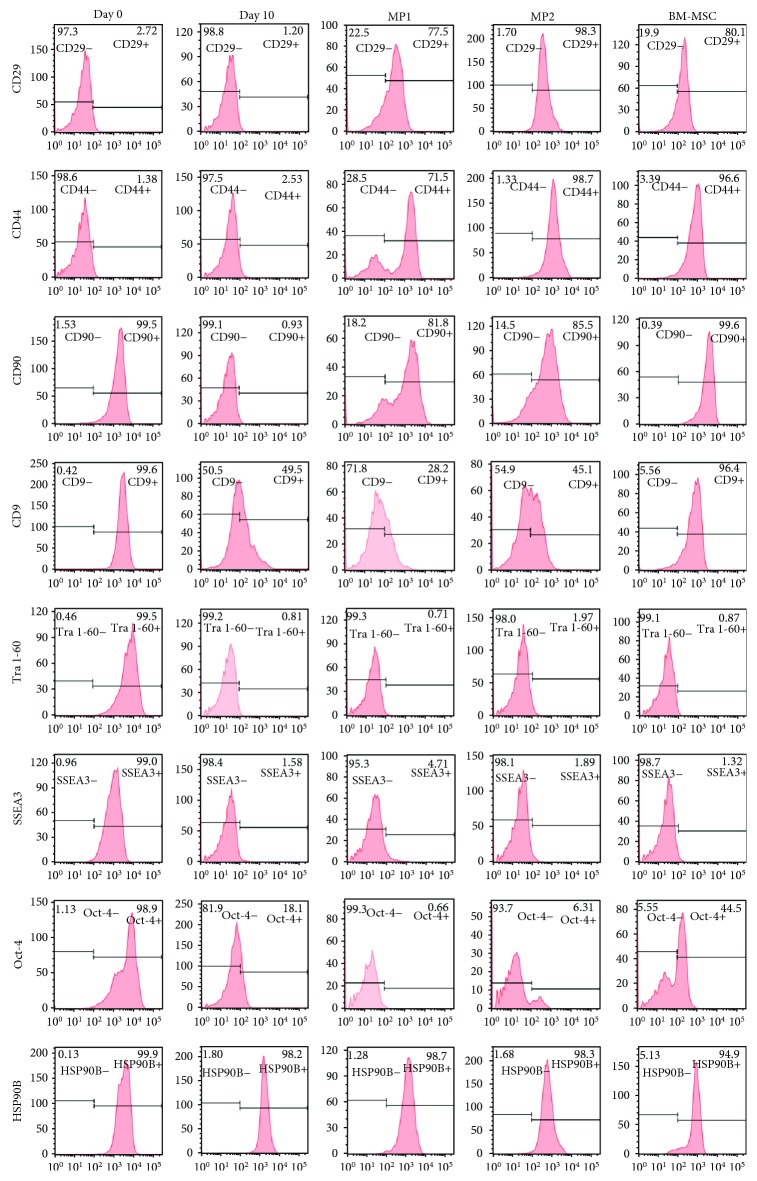
Immunophenotype of iPSCs/ESCs, BM-hMSCs, and iPSC/ESC-derived MSCs before and during the differentiation process. Shown are representative flow cytometry histograms of iPSCs/ESCs, BM-hMSCs, and iPSC/ESC-derived MSCs stained for CD44, CD29, CD90, CD9, Tra 1-60, SSEA-3, Oct-4, and HSP90*β*.

**Figure 3:**
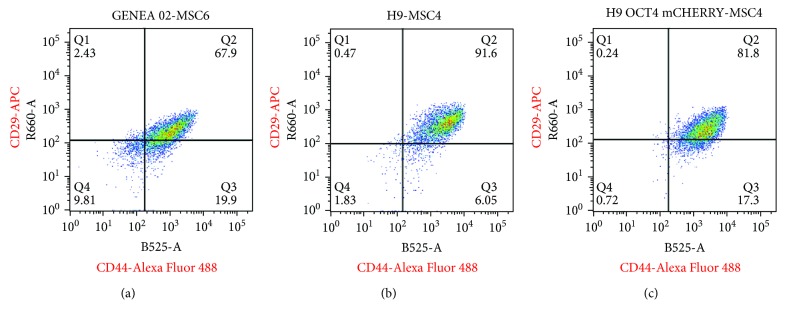
Flow cytometry dot plots showing the coexpression of CD44 and CD29 in GENEA 02-MSC6 (a), H9-MSC4 (b), and H9 OCT4 mCHERRY-MSC4 (c) cells. The gates denoting positive expression of CD44 and CD29 were selected by exclusion of greater than 99% of events appearing in the isotype control pattern. CD44 and CD29 were found to be strongly coexpressed in both iPSC- and ESC-derived MSC lines examined. Each plot represents 10.000 FACS events.

**Figure 4:**
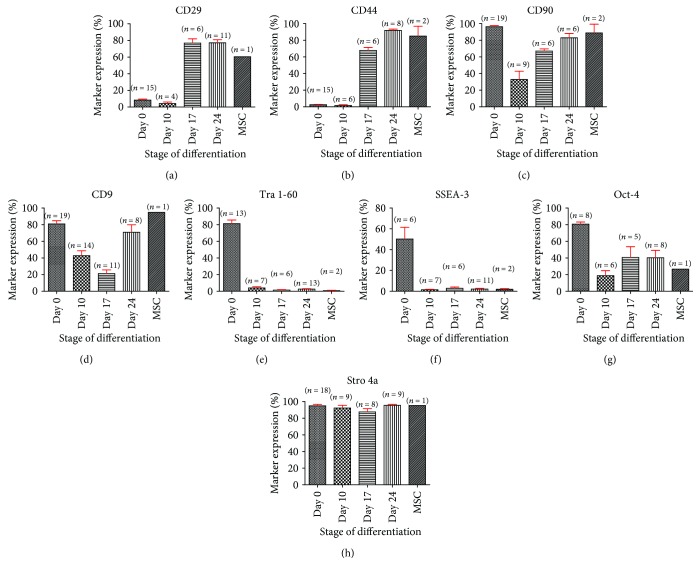
Average CD29 (a), CD44 (b), CD90 (c), CD9 (d), Tra 1-60 (e), SSEA-3 (f), Oct-4 (g), and HSP90*β* (h) expression in BM-hMSCs and iPSC/ESC-derived MSCs before and during the mesengenic differentiation process. The results suggest that both CD44 and CD29 lacked expression in undifferentiated cells and cells at MP0. The expression of these markers only increased to levels comparable to those of BM-hMSCs after the first passage into MPC Growth MEM media. By MP2, the expression of CD44 increased again while the expression of CD29 remained at the same level. CD90, CD9, and HSP90*β*, however, are highly expressed in both undifferentiated and mesenchymal stem cells derived from the bone marrow. HSP90*β* remained strongly expressed during the entire mesengenic differentiation process while the expression of CD90 and CD9 decreased after 10 days of exposure to SB431542 and increased again by MP1 and MP2, respectively. By MP2, the expression of CD90, CD9, and HSP90*β* reached levels comparable to those of BM-hMSCs. Lastly, Tra 1-60, SSEA-3, and Oct-4 are only strongly expressed in undifferentiated cells while BM-derived MSCs and ESC/iPSC-derived MSCs lacked expression of these pluripotent stem cell markers. Both Tra 1-60 and SSE-3 remained poorly expressed during the entire mesengenic differentiation process. On the other hand, the expression of Oct-4 decreased after 10 days of exposure to SB431542 and increased slightly after the first passage in MPC Growth MEM media, remaining at this level on subsequent passages. By MP2, the expression of Tra 1-60, SSEA-3, and Oct-4 remained at levels comparable to those of BM-hMSCs. An average of the expression of these markers in all cell lines examined before and during the differentiation process was combined to construct the graphs.

All pluripotent stem cells examined lacked expression of typical hMSC markers CD29 and CD44 before and after 10 days of treatment with SB431542. Furthermore, the expression of CD44 and CD29 increased during the differentiation protocol after the first passage in MPC Growth MEM media to levels comparable to those of BM-derived human MSCs (Figure 2). Flow cytometric analysis also revealed that most cells were positive for both CD44 and CD29 after the differentiation process (Figure 3). CD90, CD9, and HSP90*β*, however, were highly expressed by all pluripotent stem cells tested before the differentiation process. Specifically, the expression of CD90 decreased sharply after 10 days of exposure to SB431542, started to increase again at MP1, and reached levels comparable to those of BM-hMSCs by MP2 while the expression of CD9 decreased sharply after 10 days of exposure to SB431542 and started to increase again only by MP2. HSP90*β* remained strongly expressed during the entire differentiation process (Figure 2). Lastly, Tra 1-60, Oct-4, and SSEA-3 were also strongly expressed before the differentiation process. After 10 days of exposure to SB431542, however, the expression of Tra 1-60, Oct-4, and SSEA-3 decreased sharply and remained at low levels by MP1 and MP2 (Figure 2). An average of the expression of these markers in all cell lines examined before and during the differentiation process was combined to construct the graphs observed in Figure 4.

### 3.3. Quantitative Real-Time PCR Analysis

**Figure 5:**
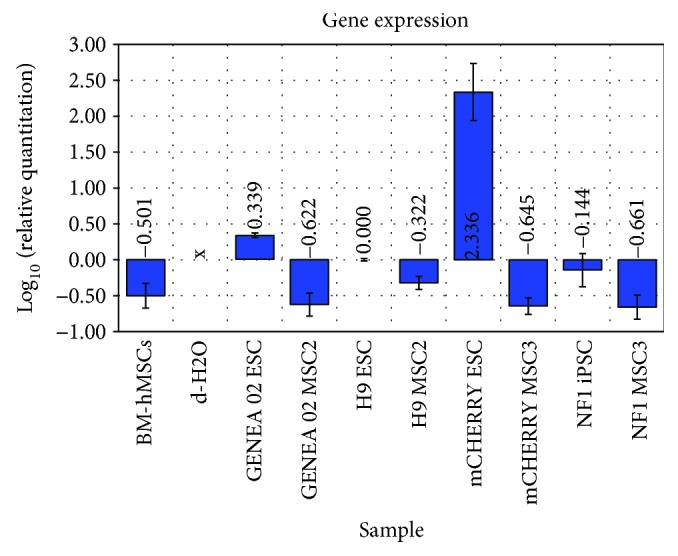
Oct-4 expression analysis of undifferentiated iPSCs/ESCs, BM-hMSCs, and iPSC/ESC-derived MSCs. The expression of Oct-4 was downregulated in BM-hMSCs and iPSC/ESC-derived MSCs in comparison with the undifferentiated iPSCs and ESCs. Gene expression was normalized to 18S. Three technical replicates were performed for each sample.

The expression of the Oct-4 gene (a gene involved in maintaining pluripotency) was compared between undifferentiated cells, BM-hMSCs, and iPSC/ESC-derived MSCs. Significant differences in the expression of Oct-4 between undifferentiated iPSCs/ESCs, BM-derived MSCs, and iPSC/ESC-derived MSCs were observed by quantitative real-time PCR analysis. As expected, the expression of Oct-4 was decreased in BM-hMSCs and iPSC/ESC-derived MSCs in comparison with the undifferentiated iPSCs and ESCs (Figure 5).

### 3.4. Osteogenic Differentiation Results

**Figure 6:**
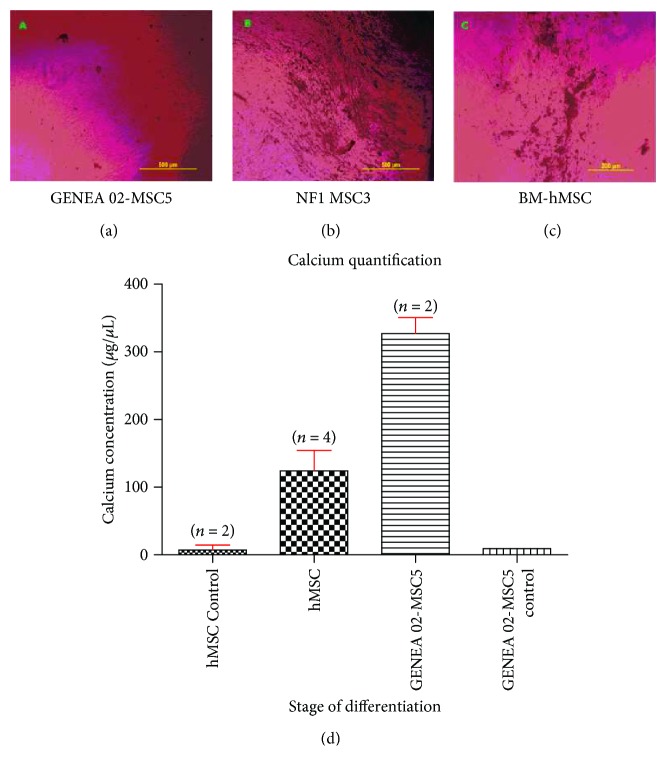
Morphological characteristics of GENEA 02-MSC5 (a), NF1-MSC3 (b), and BM-hMSCs (c) after 31 days of directed osteogenic differentiation. Both NF1-MSC3 and BM-hMSCs displayed a strong osteogenic differentiation ability as indicated by the presence of a mineralized matrix in the culture. GENEA 02-MSC5 cells, however, showed a limited osteogenic differentiation ability when compared with NF1-MSC3 cells. (d) Average calcium concentration in BM-hMSCs and ESC-derived MSCs after 31 days of culture in osteogenic differentiation media or MPC Growth MEM media. A higher concentration of calcium was observed in GENEA 02-MSC5 and BM-derived MSCs after 31 days of directed osteogenic differentiation when compared with the concentration of calcium detected in GENEA 02-MSC5 and BM-hMSCs (negative controls) cultured in MPC Growth MEM media during the same period of time.

To examine the ability of iPSC/ESC-derived MSCs to differentiate to osteocytes, BM-hMSCs, GENEA 02-MSC5 cells, and NF1-MSC3 cells were subjected to standard osteogenic differentiation conditions. Twenty-five days after culture in osteogenic differentiation media, GENEA 02-MSC5, NF1-MSC3, and BM-hMSCs displayed robust osteogenic differentiation as indicated by the presence of a mineralized matrix in the culture. Furthermore, an extremely high concentration of calcium was observed in GENEA 02-MSC5 cells after 31 days of directed osteogenic differentiation even when compared with the concentration of calcium detected in BM-derived MSCs at the same conditions (Figure 6).

### 3.5. Adipogenic Differentiation Results

**Figure 7:**
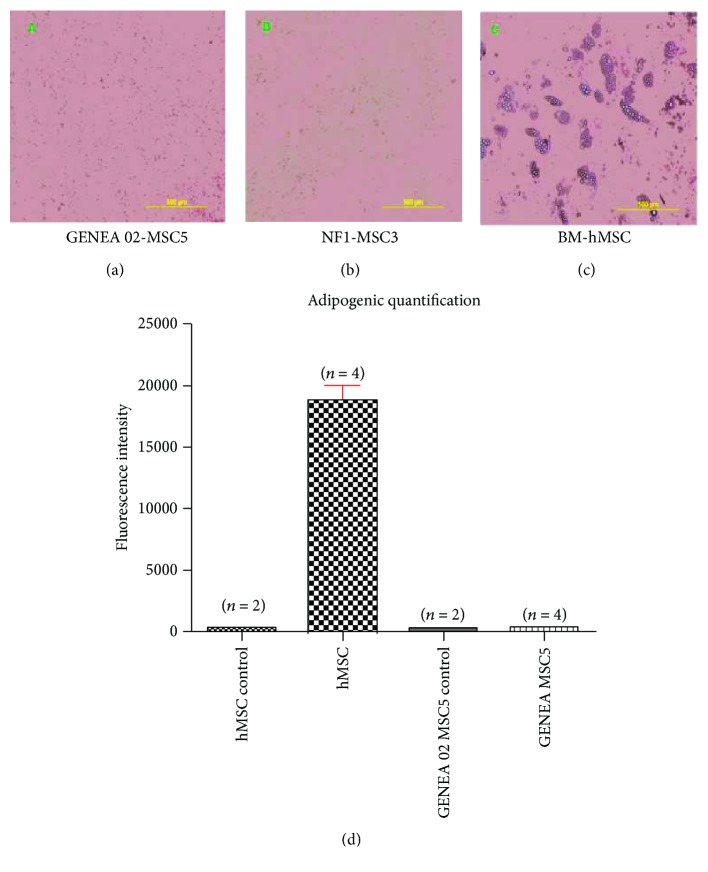
Morphological characteristics of GENEA 02-MSC5 (a), NF1-MSC3 (b), and BM-hMSCs (c) after 28 days of directed adipogenic differentiation. No signs of adipogenic differentiation were observed in GENEA 02-MSC5 and NF1-MSC3 cells after 28 days of directed adipogenic differentiation. The presence of lipid-rich vacuoles within cells was only observed in BM-derived MSCs. (d) Average fatty acid concentration in BM-hMSCs and ESC-derived MSCs after 28 days of culture in adipogenic differentiation/maintenance media or MPC Growth MEM media. A higher fatty acid synthesis was only observed in BM-derived MSCs after 28 days of directed adipogenic differentiation when compared with the concentration of calcium detected in GENEA 02-MSC5 cells subjected to standard adipogenic differentiation and GENEA 02-MSC5 and BM-hMSCs (negative controls) cultured in MPC Growth MEM media during the same period of time.

To examine the ability of iPSC/ESC-derived MSCs to differentiate to adipocytes, BM-hMSCs, GENEA 02-MSC5 cells, and NF1-MSC3 cells were subjected to standard adipogenic differentiation conditions for 28 days. No signs of adipogenic differentiation were observed after the osteogenic differentiation process in GENEA 02-MSC5 cells and NF1-MSC3 cells. After 28 days of directed adipogenic differentiation, only BM-derived MSCs displayed robust adipogenic differentiation as indicated by the presence of lipid-rich vacuoles within cells. Furthermore, an increase in the fatty acid concentration was only observed in BM-derived MSCs after 28 days of directed adipogenic differentiation (Figure 7).

## 4. Discussion

In this study, the objective was to determine if pluripotent stem cells exposed to SB431542, a TGF-*β* inhibitor, are able to differentiate to mesenchymal stem cells, judging by morphology, expression of mesenchymal and pluripotent stem cell markers, expression of pluripotency-related genes, and ability to differentiate to osteocytes and adipocytes. Directed differentiation will probably be the most important technique used during potential future use of human ESCs and iPSCs for therapy or research in order to obtain enriched populations of cell types of interest. The advantages of generating MSCs from human iPSCs and ESCs include the elimination of the necessity for a new bone marrow donation once the cells reach senescence and the generation of a more homogeneous population of hMSCs for therapy with a higher proliferative ability and possibly without the risk of forming teratomas. Furthermore, it is possible that hMSCs derived from a pluripotent stem cell line can also be used to stimulate engraftment of other cells derived from the same cell line in future therapies.

### 4.1. Analysis of Cell Morphology and Immunophenotypic Characteristics

This study showed that the exposure of iPSCs and ESCs to SB431542 promotes the differentiation of these pluripotent stem cells to cells with characteristics that closely resemble those of hMSCs. In the absence of SB431542, undifferentiated iPSCs and ESCs cultured on Geltrex in complete E8 medium appeared as circular and flat colonies with a well-defined border. As expected, these cells lacked expression of the mesenchymal stem cell markers CD29 and CD44 and expressed high levels of the pluripotent stem cell markers Tra 1-60, Oct-4, SSEA-3, CD90, CD9, and HSP90*β*.

After 10 days of exposure to SB431542, both iPSCs and ESCs adopted a cuboidal epithelial-like morphology with colonies of differentiated cells organized in a circular conformation. SB431542 is a known TGF pathway inhibitor [34] capable of stimulating the differentiation of pluripotent stem cells into a number of cell types, including neural [35], retinal [36], hematopoietic [37], and endothelial [38] cells. SB431542 blocks SMAD2/3 phosphorylation and nuclear translocation by inhibiting the activation of the transforming growth factor-beta superfamily type I activin receptor-like kinase (ALK) receptors ALK4, ALK5, and ALK7 [34]. Pluripotent stem cells exposed to SB431542 lacked expression of both mesenchymal stem cell markers CD29 and CD44 and pluripotent stem cell markers Tra 1-60, Oct-4, SSEA-3, CD90, and CD9. It was theorized that the lack of expression of both mesenchymal and pluripotent stem cell markers is a result of the high cellular heterogeneity observed after 10 days of exposure to SB431542. HSP90*β*, however, remained strongly expressed in both iPSCs and ESCs after exposure to SB431542 due to the fact that heat shock protein 90 is one of the most common of the heat-related proteins, expressed in several types of cells. Therefore, their levels are not affected by the high cellular heterogeneity observed after 10 days of exposure to SB431542.

After seven days of culture in 10% FBS-MPC Growth media (MP1), most of the pluripotent stem cell lines analysed adopted an intermediary morphology. Despite the fact that GENEA 02, H9, and NF1-derived MSCs displayed the characteristic spindle-shaped morphology of hMSCs, BM-MSCs were still markedly larger and more elongated than all cell lines analysed by MP1. On the other hand, H9 OCT4 mCHERRY-derived MSCs exhibited a cuboidal epithelial-like morphology by MP1. Furthermore, most of the pluripotent stem cell lines analysed lacked expression of the pluripotent-specific markers SSEA-3, Tra 1-60, and Oct-4 while the expression of the mesenchymal stem cell markers CD29 and CD44 was significantly higher. Interestingly, H9 OCT4 mCHERRY cells expressed higher levels of Oct-4 compared with all other cell lines analysed by MP1. It was theorized that the higher expression of Oct-4 observed in H9 OCT4 mCHERRY cells by MP1 is responsible for the cuboidal epithelial-like morphology exhibited by these cells as Oct-4 is a protein involved in maintaining pluripotency. As the markers CD90 and HSP90*β* are commonly expressed by both pluripotent stem cells and mesenchymal stem cells, a strong expression of both markers was observed by MP1.

After two weeks of culture in 10% FBS-MPC Growth media (MP2), most of the cell lines examined (H9, GENEA 02, and NF1 cells) differentiated into spindle-shaped fibroblast-like cells with morphology similar to that of BM-MSCs. However, H9 OCT4 mCHERRY-derived MSCs were still exhibiting an intermediary morphology similar to the morphology displayed by H9, GENEA 02, and NF1-derived MSCs by MP1. The theory that the strong expression of Oct-4 in H9 OCT4 mCHERRY-derived MSCs is responsible for the cuboidal epithelial-like morphology observed by MP1 was supported by the lower expression of Oct-4 detected in this cell line by MP2. The typical spindle-shaped fibroblast-like morphology similar to that of BM-MSCs was only observed in H9 OCT4 mCHERRY-derived MSCs after three weeks of culture in 10% FBS-MPC Growth media (MP3). Furthermore, all pluripotent stem cell lines analysed were still negative for the pluripotent-specific markers SSEA-3, Tra 1-60, and Oct-4 and strongly positive for the expression of the mesenchymal stem cell markers CD29 and CD44 by MP2. It was also demonstrated that both CD29 and CD44 were coexpressed in all ESC-derived MSC lines examined. In addition, CD90 and HSP90*β* were still highly expressed in all cell lines analysed while the expression of CD9 (a marker commonly expressed by both pluripotent stem cells and mesenchymal stem cells) increased to levels comparable to that of BM-derived MSCs in GENEA 02 and NF1-derived MSCs. On the other hand, the expression of the pluripotent stem cell marker Oct-4 increased slightly in NF1 cells by MP2, indicating a possible reversion to pluripotency in iPSC-derived MSC lines obtained by this method. The notion that differentiated human iPSCs are able to revert to a pluripotent phenotype was demonstrated by Polanco et al. [39]. This study demonstrated that two of the iPSC lines examined exhibited evidence of reversion to a pluripotent phenotype once differentiated while the ESC line did not. Controversially, it was also demonstrated by Polanco et al. [39] that NF1 cells are not able to revert to a pluripotent state once differentiated. Therefore, iPSC-derived MSC lines should be tested in future experiments for the expression of other pluripotency-related genes and cell markers and ability to form teratomas in order to assess the safety of a possible clinical use of MSCs derived from iPSCs.

### 4.2. Analysis of Oct-4 Gene Expression

Furthermore, the pluripotency-related gene Oct-4 was found to be strongly downregulated in all iPSC- and ESC-derived MSC lines examined by MP2/MP3, when the cells adopted a MSC-like morphology and immunophenotype. The mRNA levels of Oct-4 determined by the qPCR array correlated empirically to protein expression determined by flow cytometry in most of the cell lines examined. Despite the fact that a strong expression of Oct-4 was detected by FACS analysis in NF1-derived MSCs, the qPCR data obtained showed a downregulation of the Oct-4 gene when compared with all undifferentiated pluripotent stem cell lines. It was theorized that the downregulation of the Oct-4 mRNA concurrent with the upregulation of the Oct-4 protein may be a result of a disruption in some component involved with the Oct-4 normal turnover or a stabilization of Oct-4 through protein-protein interactions. Furthermore, the accumulation of the Oct-4 protein may act as a repressor of transcription, signaling that there is no need to synthesize Oct-4 since it is already expressed in certain amounts.

### 4.3. Differentiation Potential Assessment

Finally, it was also possible to conclude that both iPSC- and ESC-derived MSC lines displayed more limited adipogenic differentiation compared with their osteogenicity. After 32 days of culture in osteogenic differentiation media, the presence of a mineralized matrix in the culture was observed in both GENEA 02- and NF1-derived MSCs. Furthermore, a significantly higher concentration of calcium was observed in GENEA 02-derived MSC culture in osteogenic differentiation media for 32 days when compared with the same cell line maintained in 10% FBS-MPC Growth media during the same period of time. The amount of calcium detected in GENEA 02-derived MSCs after 32 days of culture in osteogenic differentiation media was even higher than the amount of calcium detected in BM-hMSCs under the same conditions. Due to technical issues, the concentration of calcium in NF1-derived MSCs could not be assessed. It is possible to speculate that these cells are also producing high levels of calcium based on the images obtained under the microscope that demonstrated the presence of a large mineralized matrix in the NF1-derived MSCs maintained in osteogenic differentiation media for 32 days. On the other hand, no sign of adipogenic differentiation was observed in any iPSC/ESC-derived MSC line after four weeks of directed adipogenic differentiation. The presence of lipid-rich vacuoles within cells was only observed in BM-derived MSCs. In addition, a high synthesis of fatty acids was also only observed in BM-derived MSCs while the levels of fatty acids detected in GENEA 02-derived MSCs were comparable to those of both BM-MSCs and GENEA 02-MSCs maintained in 10% FBS-MPC Growth media during the same period of time.

### 4.4. Considerations for Future Studies

Several studies are currently being undertaken in order to develop methods to differentiate pluripotent stem cells towards specific lineages [40–42]. These methods are of huge interest as the cells obtained might be used for a number of clinical and nonclinical future applications. Among them, some studies described successful generation of MSC-like cells derived from the differentiation of pluripotent stem cells. For instance, Stojkovic et al. [43] demonstrated that fibroblast-like cells can be spontaneously produced in human ESC cultures. The expression of typical MSC cell surface markers such as CD44 and CD90 was also detected in these cells. Furthermore, the generation of MSC-like cells derived from the differentiation of pluripotent stem cells was subsequently achieved in a study performed by Barberi et al. [44]. In this study, cells expressing the typical mesenchymal stem cell markers CD29, CD44, CD73, and CD105 and with the potential to differentiate into osteocytes, chondrocytes, and adipocytes were obtained after 40 days of coculturing human ESCs with OP9 cells.

Interestingly, MSC-like cells generated by distinct methods do not express the exact same phenotypic characteristics. Even minor differences in the culture methodology can result in minor or major differences in the phenotype of differentiated cells. For example, elevated levels of the typical MSC markers CD73 and CD105 were found to be expressed by the MSC-like cells obtained by Olivier et al. [45]. However, a high expression of SSEA-4 (a typical pluripotent stem cell marker) was also observed in these cells. Furthermore, MSC-like cells obtained by Hwang et al. [46] expressed high levels of CD29 and CD105, but lacked CD73, a marker typically expressed by BM-derived MSCs. On the other hand, the method described in this study was able to generate iPSC- and ESC-derived MSCs that express high levels of cell surface markers commonly expressed by BM-derived MSCs such as CD29, CD44, CD90, and CD9 and lacked expression of the pluripotency-related markers SSEA-3, Tra 1-60, and Oct-4. It will be important for the development of future therapies to assess how such immunophenotypic differences could impact the functional characteristics of iPSC- and ESC-derived MSCs. Despite these potential issues, ESC-derived MSCs have been successfully used to treat some diseases in animal models. For instance, Wang et al. [47] demonstrated that the use of human ESC-derived MSC outperforms BM-derived MSCs in the treatment of multiple sclerosis in a mouse experimental model.

In summary, multiple methods have tried to reach the most efficient and direct generation of iPSC- and ESC-derived MSCs. Some studies have chosen the EB method while others omitted this step; some of them used a coculture while others maintained cells in feeder-free conditions in multiple media formulations with and without serum. Despite the fact that these studies have contributed immensely to the field, it is an absolute necessity to reach a consensus on the most appropriate method for the generation of MSCs derived from pluripotent stem cells in order to make therapies based on the use of iPSC-MSCs and ESC-MSCs a reality.

## 5. Conclusion

The results obtained showed that it is possible to generate MSC-like cells from both iPSCs and ESCs. In general, mesenchymal stem cells derived from the differentiation of iPSCs and ESCs displayed a MSC-like morphology, expressed high levels of mesenchymal stem cell markers, and lacked expression of pluripotent-specific markers. Furthermore, the expression of the pluripotency-related gene Oct-4 was downregulated in both iPSCs and ESC-derived MSCs. Despite the fact that an adipogenic differentiation could not be reached in MSCs derived from pluripotent stem cells, a strong osteogenic potential was observed in both iPSC- and ESC-derived MSCs. The generation of MSCs from pluripotent stem cells represents a promise for the future of tissue engineering and regenerative medicine.

The method described in this study proved to be an efficient system for generating MSC-like cells from human ESCs and iPSCs. However, several studies still need to be conducted in order to determine whether mesenchymal stem cells derived from this method are clinically applicable or not. For instance, it is imperative to determine if mesenchymal stem cells derived from this method are able to spontaneously return to pluripotency. If the mesenchymal stem cells derived from this method do indeed spontaneously return to a pluripotent phenotype once differentiated, there may be serious implications for the safety and practicality of future therapies involving these cells. Furthermore, it is also important to assess the ability of both iPSC- and ESC-derived MSCs to form teratomas and the presence of any karyotypic abnormalities before they can be clinically used as a reliable source of mesenchymal stem cells for therapy. Despite these potential issues, it is hoped that eventually the generation of mesenchymal stem cells from pluripotent stem cells will be achieved and iPSC/ESC-derived MSCs will be safe to use for future stem cell therapies.
